# Research on the recognition model of exercise fatigue based on the fusion of sEMG and ECG signals

**DOI:** 10.1016/j.isci.2024.109365

**Published:** 2024-02-29

**Authors:** Hao Li, Dujuan Li

**Affiliations:** 1 School of Sports Medicine and Rehabilitation, North Sichuan Medical College, Nanchong 637100, China; 2 School of Sports Medicine and Rehabilitation, North Sichuan Medical College, Nanchong 637100, China

**Keywords:** Health sciences, Medicine, Medical specialty, Internal medicine, Cardiovascular medicine, Natural sciences, Biological sciences, Biology experimental methods

## Abstract

This study significantly enhances the accuracy of exercise state identification in wearable devices through improved denoising techniques for sEMG and ECG signals. By adopting an optimized Variational Mode Decomposition (VMD) method, combined with the Improved Sparrow Search Algorithm and Second Generation Wavelet Transform (ISSA-VMD-SWT), and introducing chaos mapping to strengthen the algorithm’s initial population, this approach effectively reduces noise while preserving key fatigue-related features. In tests conducted on data from 32 participants, the method achieved accuracy rates of 93.25%, 95.16%, and 93.05% for identifying “Easy,” “Transition,” and “Tired” exercise states, respectively, showing significant advantages over traditional denoising techniques. These results indicate that the denoising technology developed in this study represents a significant technological advancement for the application of ECG and sEMG fatigue identification technologies in wearable health monitoring devices.

## Highlights

• Proposes an enhanced sparrow search algorithm with second-gen wavelet transform
• Overcomes slow convergence and local solution issues, enhancing denoising effect
• Proposed method enhances denoising, leading to higher accuracy in fatigue recognition model

## Graphical abstract

**Figure:**
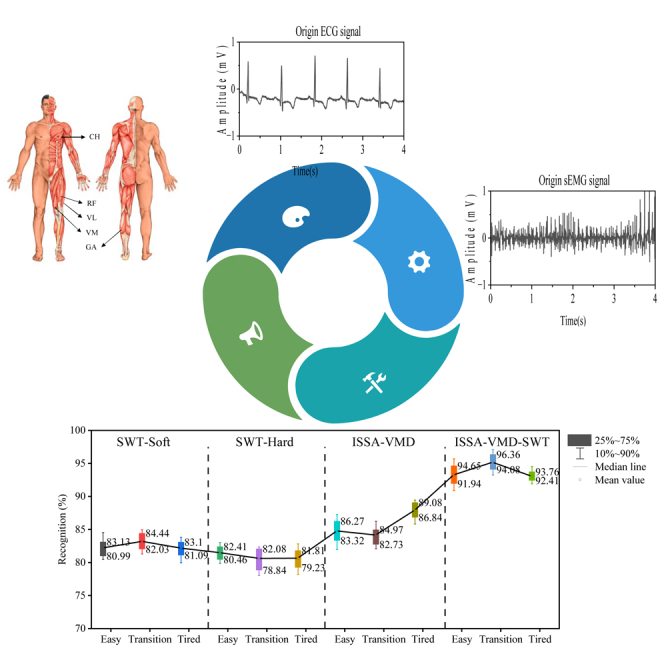

## Introduction

Muscle fatigue is defined as the decrease in the efficiency of action potential propagation between the sarcolemma and muscle fibers, resulting from the accumulation of lactate, hydrogen ions, and inorganic phosphates in the blood during exercise, leading to a decrease in muscle pH, maximum voluntary contraction capacity, and maximum power output of the muscle.^1^ Accurate identification of muscle fatigue status is of great significance in preventing sports injuries and has important implications in the fields of kinematics and rehabilitation medicine. Non-invasive detection techniques can be employed for precise identification of muscle fatigue, offering broad application prospects.^2^ These techniques primarily include surface electromyography (sEMG),^3^ sonomyography (SMG), near-infrared spectroscopy (NIRS) analysis,^4^ and Mechanomyography (MMG).^5^ In recent years, there has been increasing attention from researchers on the combined use of sEMG and electrocardiogram (ECG) for the identification of muscle fatigue status. These two signals respectively represent the superficial EMG of the skin and the sustained depolarization of myocardial cells, both containing rich information on human neural function. The combination of these two methods can provide a highly accurate and robust approach for identifying muscle fatigue status.

When applying these methods, the first challenge is to address the denoising issue in weak bioelectrical signals of the human body. Noise in sEMG and ECG signals is considered as one of the crucial factors that affect the recognition performance. Chatterjee et al. emphasized that denoising is a key preprocessing step.^6^ The sources of noise mainly include baseline drift, power line interference, muscle artifacts, and channel noise. Several researchers have conducted studies on denoising weak bioelectrical signals, employing methods such as empirical mode decomposition (EMD), ensemble empirical mode decomposition (EEMD), improved empirical mode decomposition, and wavelet-based digital filtering. In comparison to these methods, the more recent denoising technique, variational mode decomposition (VMD), can overcome drawbacks like mode mixing and endpoint effects, making it one of the recognized effective decomposition methods for weak bioelectrical signals. Xiao et al. proposed a method that combines wavelet soft thresholding with VMD for denoising sEMG signals, where the key parameters of VMD were selected using an empirical approach. Comparative results demonstrated that this method outperformed Empirical Mode Decomposition and wavelet-based methods, highlighting the importance of parameter selection in VMD for achieving superior model performance.^7^ However, the selection of VMD parameters relies heavily on subjective judgment, making it less user-friendly for non-experts or sports practitioners. Therefore, many researchers have devoted significant efforts to introducing adaptive mechanisms for parameter selection in VMD.

The denoising efficacy of VMD heavily relies on the choice of penalty factors α and decomposition levels k. Swarm intelligence optimization algorithms, like Beetle Antennae Search, Gray Wolf, and Cuckoo Search, have been employed to optimize VMD parameters. Wang et al. proposed an improved VMD method based on the Beetle Antennae Search algorithm, where the kurtosis of intrinsic mode functions was used as the fitness function during the search process.^8^ Gai et al. optimized VMD parameters using a hybrid Gray Wolf algorithm, significantly improving the optimization speed.^9^ Yan et al. optimized VMD parameters using the Cuckoo Search algorithm.^10^ Numerous research findings have shown that swarm intelligence parameter optimization algorithms can indeed introduce adaptive mechanisms for parameter selection in VMD. However, swarm intelligence algorithms have drawbacks such as a tendency to get stuck in local optima and slow convergence speed, leading to different optimization results and a lack of unified denoising effects when using these algorithms to optimize the penalty factor α and k. Prior to this study, there have been few research efforts on the improvement of swarm intelligence optimization algorithms in the field of VMD for denoising muscle signals.

In this paper, the fast swarm intelligent optimization algorithm is applied to the parameter optimization of denoising algorithm, which proposes an improved denoising method called *Improved Sparrow Search algorithm to optimize VMD combined with Second Generation Wavelet Transform* (ISSA-VMD-SWT). Through the comparison of denoising effects on simulated noisy ECG and sEMG signals, analysis of denoising performance on actual collected signals, and the analysis of the improvement in fatigue recognition performance by combining the denoising algorithm with sEMG and ECG, it is demonstrated that ISSA-VMD-SWT can effectively handle both local and global characteristics of the signals, making it one of the suitable methods for preprocessing muscle signals. The overall arrangement of this paper is as follows: the second part presents the materials and methods, including data collection and a detailed description of ISSA-VMD-SWT. The third part compares the denoising effects on simulated noisy signals and real collected signals, as well as the analysis of the improvement in muscle fatigue recognition using the proposed denoising method. The fourth part presents the conclusions. This paper will provide a reliable and efficient denoising method for the joint use of sEMG and ECG, supporting the development and promotion of wearable devices in the field of sports science in the future.

## Result

### ECG signal characteristics analysis under different fatigue states

Due to the significant variations in ECG signals caused by individual and environmental differences, Heart Rate Variability (HRV) analysis is employed in this study to analyze the ECG signals. HRV provides a comprehensive set of evaluation metrics derived from the ECG. It can serve as a reference for assessing mental and physiological stress.^11^ This paper utilizes several time-domain and frequency-domain HRV indices. The time-domain indices include the mean of normal to normal intervals (NN.mean) and the proportion of NN50 divided by the total number of NNs (PNN50). The frequency-domain indices include the total power (TP) and the High frequency from 0.15 Hz to 0.4 Hz (HF). The characteristic parameters of HRV are shown in Table 1.

**Table 1:** The statistical analysis of HRV feature parameters under different levels of muscle fatigue

Indicator	Formula or Frequency band	Easy		Transition		Tried	
		Mean	Var	Mean	Var	Mean	Var
NN.mean	∑i=1NNNiN	0.875	0.010	0.839	0.012	0.794	0.013
rMSSD	$sqrt{(mean\left(NN_{i+1}-NN_{i}\right)}^{2})$	0.249	0.049	0.254	0.051	0.266	0.052
PNN50	count(|NNi+1|−NNi)>50msN−1	0.487	0.022	0.306	0.019	0.222	0.014
TP	0.003 Hz–0.4 Hz	1.521	8.254	1.828	11.372	2.641	19.291
HF	0.15 Hz–0.4 Hz	0.354	0.524	0.521	0.865	0.771	1.590
LF	0.04 Hz–0.15 Hz	1.021	3.254	1.168	5.426	1.679	9.356
VLF	0.003 Hz–0.04 Hz	0.101	0.010	0.138	0.017	0.191	0.043
LF/HF	LF/HF	1.255	0.254	1.521	0.603	1.860	1.171

The "mean" represents the average value, "var" denotes the variance.

HRV exhibits different patterns under three different states. (1) For the parameter NN.mean, the average value is higher in the Easy and Transition states, but slightly lower in the Tired state. However, the variance increases, indicating greater fluctuations in NN.mean during the Tired state. (2) rMSSD is used to evaluate the degree of heart rate variation between adjacent heartbeats, reflecting the rapid or short-term changes in HRV. The calculated values for rMSSD show slight differences but with minimal changes. The values are generally close to each other. (3) PNN50 represents the percentage of adjacent RR intervals differing by more than 50 ms. In the Easy state, PNN50 has a higher value, while in the Transition and Tired states, the value is lower. The low variance also indicates less variability in this parameter. This suggests a higher stability in the autonomic nervous system regulation of the participants. (4) total spectral power gradually increases from the Easy to Tired state. This indicates that overall HRV increases with the level of muscle fatigue. (5) HF and LF values increase sequentially with increasing muscle fatigue. This suggests a greater contribution of HF and LF to HRV. (6) VLF also increases with muscle fatigue, indicating an imbalance in sympathetic and parasympathetic nervous system activity and an impact on autonomic nervous system regulation. (7) the LF/HF ratio indicates the balance between sympathetic and parasympathetic activity. When LF/HF is lower, it indicates a relatively balanced state between sympathetic and parasympathetic activity. As LF/HF increases, sympathetic activity becomes relatively enhanced while parasympathetic activity gradually weakens. These findings illustrate the diverse patterns of HRV parameters under different states of muscle fatigue.

Based on the comprehensive data analysis, the development of muscle fatigue is a relatively complex process with various changes. The sympathetic and parasympathetic nervous systems have a significant impact on ECG signals, and multiple HRV parameters reflect the individual’s sympathetic and parasympathetic responses to muscle fatigue. However, one obvious problem is that HRV parameters vary from person to person, making it impossible to determine muscle fatigue from just one or a few parameters.

### Analysis of sEMG signal characteristics under different fatigue conditions

We collected sEMG signals from the rectus femoris (RF), vastus lateralis (VL), vastus medialis (VM), and gastrocnemius (GA) muscles through experiments, and conducted an analysis on the collected data. The sEMG signals exhibited complexity, and fatigue cannot be analyzed solely based on the raw signals. Therefore, we selected commonly used sEMG signal features for analysis, including integrated electromyography (IEMG), root-mean-square (RMS), mean power frequency (MPF), and median frequency (MF). The calculation methods for these four indices can be found in ref. 12.

**Figure 1:**
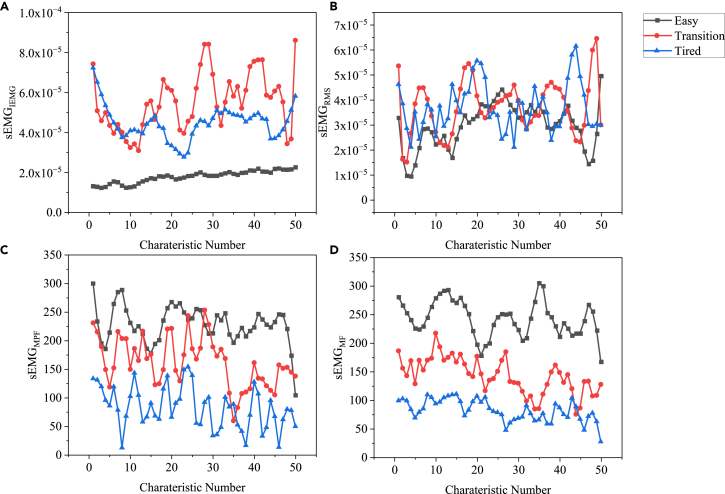
sEMG signal characteristics under different fatigue states (A) ${\text{sEMG}}_{\text{IEMG}}$. (B) ${\text{sEMG}}_{\text{RMS}}$. (C) ${\text{sEMG}}_{\text{MPF}}$. (D) ${\text{sEMG}}_{\text{MF}}$.

Figure 1 shows the sEMG signal features of RF in different fatigue states (Easy, Transition, Tired). In Figure 1A, ${\text{sEMG}}_{\text{IEMG}}$ can easily distinguish Tired and Easy states. In the Tired state, ${\text{sEMG}}_{\text{IEMG}}$ has higher values and larger variance, indicating greater fluctuations. In contrast, in the Easy state, ${\text{sEMG}}_{\text{IEMG}}$ has smaller values and less variability. Therefore, ${\text{sEMG}}_{\text{IEMG}}$ exhibits good discriminative ability and reflects the overall muscle activation level, where higher values correspond to stronger muscle stimulation. In Figure 1B, ${\text{sEMG}}_{\text{RMS}}$ is mainly used to quantify the amplitude characteristics and reflects the level of muscle contraction force. The results show that in the Tired state, ${\text{sEMG}}_{\text{RMS}}$ amplitude increases because more neural activation is required to maintain the same force output during fatigue. However, the separation of RMS between Tired and Easy states is not significant, indicating that this parameter cannot accurately reflect the degree of muscle fatigue. In Figure 1C, ${\text{sEMG}}_{\text{MPF}}$ represents the median frequency, which plays a role in reflecting muscle fatigue level and fiber type. According to the results, the MPF decreases when the body is in a fatigued state. This is because as fatigue progresses, the activation pattern of muscle fibers changes, leading to an increase in low-frequency information. In the Easy stage, the MPF value is relatively high, indicating a more normal activation pattern of muscle fibers, and the signal tends to contain higher-frequency information. The variation in MPF between individuals may differ depending on the different exercise states. In Figure 1D, ${\text{sEMG}}_{\text{MF}}$ shows a clear pattern with changes in exercise states. As the exercise continues, ${\text{sEMG}}_{\text{MF}}$ gradually decreases. This is because muscle contractions become less coordinated and the activation pattern changes. The signal shifts toward lower frequencies, resulting in a gradual decrease in ${\text{sEMG}}_{\text{MF}}$ values.

By analyzing the HRV parameters of ECG and the parameters of sEMG, such as IEMG, RMS, MPF, and MF, the body experiences exercise-induced fatigue during physical activity. This fatigue is mainly characterized by a decrease in the stability of the sympathetic and parasympathetic nervous systems and an increase in the low-frequency component of muscle signals. These findings provide a basis for a deeper understanding of ECG and sEMG signals and the development of suitable denoising methods.

## Discussion

### Comparison of different optimization algorithms

**Figure 2:**
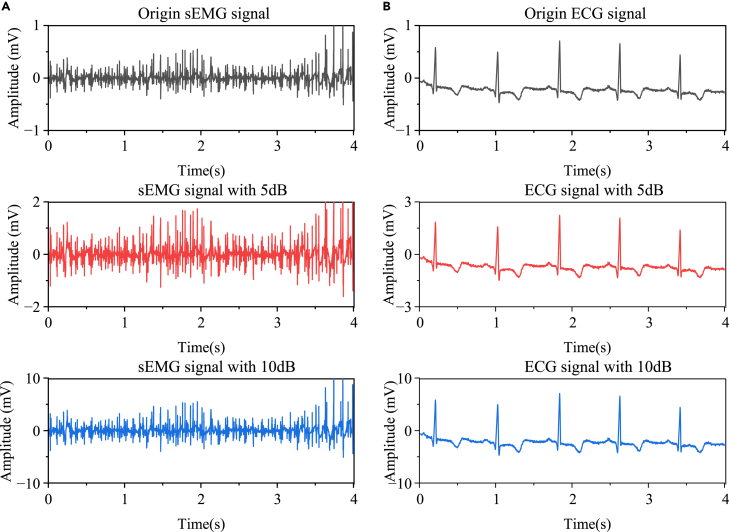
Raw signal and noisy signal (A) ECG signal, (B) sEMG signal.

To evaluate the optimization effect of ISSA on VMD parameters and the advantages of the SWT in denoising, this study used sEMG signals collected from a healthy volunteer in the NinaPro electromyography database as simulated data. The data had minimal noise and served as clean signals for experimentation. Both ECG and sEMG signals contain a significant number of low-frequency components during the Tired stage, which should be preserved while removing noise in signal processing. In the simulated signals, 5 dB and 10 dB noise signals were added. Common algorithms, including genetic algorithm (GA), particle swarm optimization (POS), and conventional sparrow search algorithm (SSA), were also used as comparisons. The parameter settings for the optimization algorithms are as follows: population size of 50, iteration count of 50, optimization range for the number of modal components [1, 2000], optimization range for the penalty factor [2, 20], safety thresholds of 0.8 for SSA and ISSA, and discoverers and informers set to 10. For GA, the crossover probability and mutation probability were set to 0.85 and 0.1, respectively. For the POS algorithm, the learning factors $C_{1}$ and $C_{2}$ were set to 1.5, and the initial and final inertia weights were set to 0.8 and 0.4, respectively. The sym8 wavelet was chosen as the basis function for the SWT, and the decomposition level was set to 3. The original sEMG and ECG signals, as well as the signals with added 5 dB and 10 dB noise, are shown in Figure 2.

**Figure 3:**
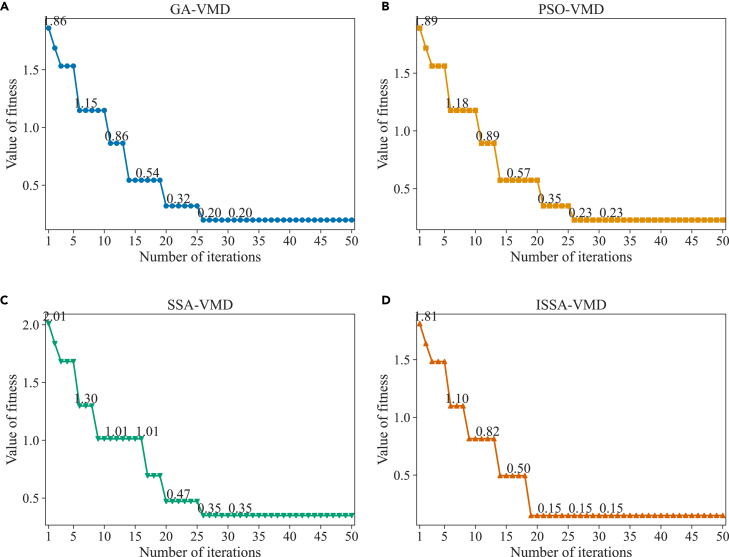
Fitness change curve (A) GA-VMD. (B) PSO-VMD. (C) SSA-VMD. (D) ISSA-VMD.

The noisy signals were input into four optimization algorithms. These algorithms were combined with VMD for decomposition, resulting in GA-VMD, PSO-VMD, SSA-VMD, and ISSA-VMD models. To reduce randomness during the optimization process, each model was run five times, and the average fitness value was obtained. The fitness variation curves of the different optimization algorithms at different iteration counts are shown in the Figure 3. The results show that at the first iteration, the initial values of the optimal $Q_{f}$ for GA-VMD, PSO-VMD, SSA-VMD, and ISSA-VMD models were slightly different, with values of 1.85, 1.88, 2.00, and 1.81. ISSA-VMD had the lowest initial value among them. These differences in initial values are related to the optimization strategies for the initial population in each algorithm. ISSA uses an improved Tent chaotic mapping to generate sequences, and then maps the sequences to different individuals to improve the distribution of the initial population. Furthermore, the $Q_{f}$ values of the four methods varied between 2.01 and 0.15, and the convergence was achieved in less than 30 iterations for all models. This indicates that all four models are effective and can be used to optimize the parameters of VMD.

The $Q_{f}$ of VMD in the four models decreases as the iteration count increases. In the initial stage of the models, $Q_{f}$ decreases rapidly. Specifically, in the GA-VMD model, $Q_{f}$ decreases to 1.15 after 6 iterations and reaches convergence at 0.2 after 26 iterations. In the PSO-VMD model, $Q_{f}$ is 0.89 after 11 iterations and reaches 0.23 after 28 iterations, achieving convergence. In the SSA-VMD model, the initial $Q_{f}$ is relatively high at 2.01. After 9 iterations, $Q_{f}$ is 1.01, and after 20 iterations, $Q_{f}$ is 0.47. Finally, $Q_{f}$ is 0.35 after 26 iterations. In the ISSA-VMD model, $Q_{f}$ rapidly decreases to 0.5 after 14 iterations, and the model converges and stabilizes after 19 iterations with a final $Q_{f}$ value of 0.15. The ISSA-VMD model is the fastest in terms of convergence speed among the four models and exhibits the best $Q_{f}$ value in terms of parameter optimization. During the experiment, we found that the convergence speed and final $Q_{f}$ of the four models are not unique, which may be related to the grid search step size adopted by each model during the optimization process. The search mechanism of the ISSA-VMD model allows it to easily escape local optima and further optimize in the search space.

### Comparison of denoising effect of denoised signal

The above comparison pertains to the parameter optimization aspect of the ISSA method. Now, we will compare the denoising performance of the established model. We will apply the ISSA-VMD method, as well as two other methods, namely the SWT with soft thresholding and the SWT with hard thresholding, to noisy ECG and sEMG signals.

**Figure 4:**
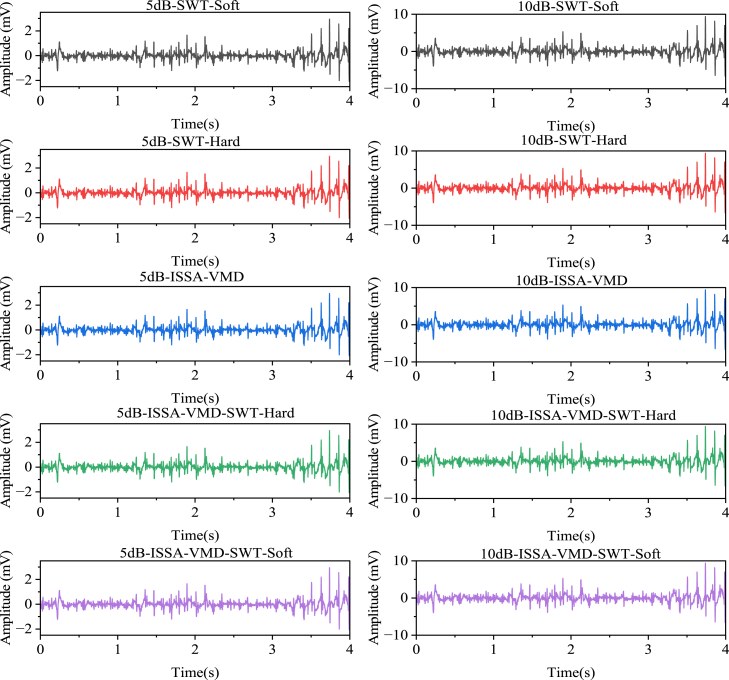
Different algorithm for noise reduction of sEMG signals with 5dB and 10dB noise 5dB indicates the denoised 5dB, 10dB indicates the denoised 10dB, Soft indicates the soft threshold, and Hard indicates the hard threshold.

Figure 4 shows the denoising effects of five methods: SWT soft thresholding, SWT hard thresholding, ISSA-VMD, ISSA-VMD with SWT soft thresholding, and ISSA-VMD with SWT hard thresholding, on sEMG signals with 5dB and 10dB noise. Ten models were established: 5dB-SWT-Soft, 5dB-SWT-Hard, 5dB-ISSA-VMD, 5dB-ISSA-VMD-SWT-Soft, 5dB-ISSA-VMD-SWT-Hard, 10dB-SWT-Soft, 10dB-SWT-Hard, 10dB-ISSA-VMD, 10dB-ISSA-VMD-SWT-Soft, and 10dB-ISSA-VMD-SWT-Hard. The evaluation metrics for each model are shown in Table 2.

**Table 2:** Denoised sEMG signal index value after denoising

Model	NCC	MSE	SNR	PE	Model	NCC	MSE	SNR	PE
5dB-SWT-Soft	0.961	0.044	10.809	0.161	10dB-SWT-Soft	0.987	0.013	15.992	0.198
5dB-SWT-Hard	0.938	0.072	8.650	0.101	10dB-SWT-Hard	0.781	0.023	13.481	0.150
5dB-ISSA-VMD	0.967	0.120	11.585	0.078	10dB-ISSA-VMD	0.991	0.062	17.539	0.133
5dB-ISSA-VMD-SWT-Soft	0.967	0.036	11.692	0.038	10dB-ISSA-VMD-SWT-Soft	0.991	0.009	17.522	0.082
5dB-ISSA-VMD-SWT-Hard	0.968	0.036	11.700	0.020	10dB-ISSA-VMD-SWT-Hard	0.991	0.994	17.535	0.069

According to the evaluation metrics of the different models after denoising, when using the SWT with soft thresholding or hard thresholding alone, the denoising results are not satisfactory. The soft thresholding method shows certain advantages over the hard thresholding method, as it achieves higher SNR values and lower MSE values. However, when comparing the PE values, the second-generation wavelet hard thresholding method performs better, with smaller peak errors. This is because the soft thresholding method, while removing noise and improving the signal-to-noise ratio, also reduces the peak values, resulting in increased peak errors between the reconstructed signal and the original signal.

When ISSA-VMD is used alone, the denoising effect is not ideal. Compared to the soft and hard thresholding methods of the second-generation wavelet, ISSA-VMD achieves better SNR, PE, and NCC, indicating that ISSA-VMD can improve the signal-to-noise ratio of the reconstructed signal while minimizing its impact on the waveform. However, the MSE value of ISSA-VMD is slightly higher than that of the soft and hard thresholding of the second-generation wavelet, indicating that using ISSA-VMD alone may not achieve a clean removal of noise details in the signal. The combination of ISSA-VMD and the second-generation wavelet in the soft and hard thresholding model shows a better overall performance. In terms of NCC and SNR, the difference between ISSA-VMD and the soft and hard thresholding is not significant, indicating that the waveform and signal-to-noise ratio after the SWT remain largely unchanged. In terms of PE, there is a noticeable decrease after the soft and hard thresholding, indicating effective correction of waveform distortion. In terms of MSE, ISSA-VMD-SWT-Hard achieves good results. Overall, when processing sEMG signals, using ISSA-VMD first helps protect the original signal waveform and suppress distortion in the reconstructed signal, and then using hard thresholding emphasizes the protection of signal details, leading to better noise suppression and higher similarity to the original signal. However, we also observed that the difference between soft and hard thresholding is very small, and it is difficult to determine the absolute advantage through a single experiment. Nevertheless, by combining the advantages of different methods, employing a strategy that combines multiple methods can achieve better results in denoising tasks.

**Figure 5:**
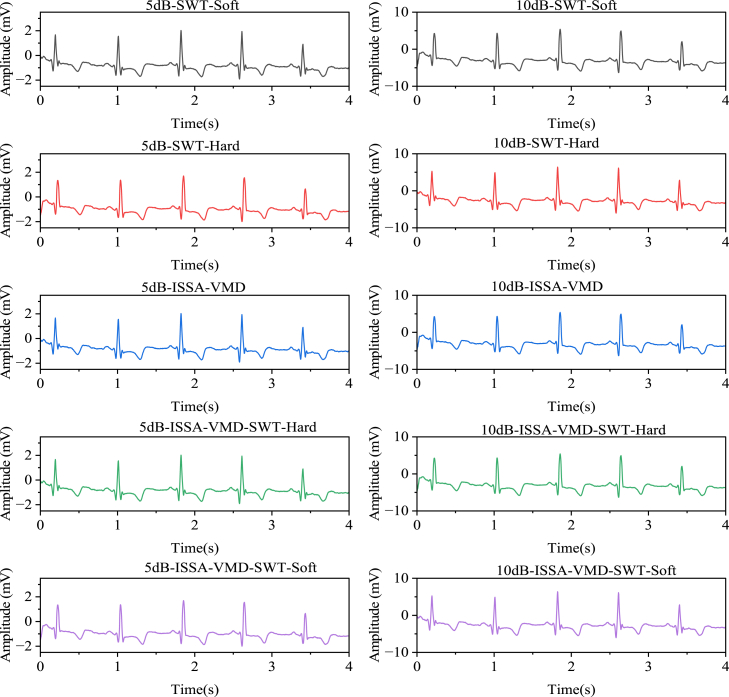
The figure shows the denoising effects of the algorithm on 5dB and 10dB noisy sEMG signals “5dB” indicates the sEMG signal with 5dB noise, and “10dB” indicates the sEMG signal with 10dB noise. “SWT” represents second-generation wavelet decomposition, “Soft” represents soft thresholding, and “Hard” represents hard thresholding.

Figure 5 shows the denoising effects of five methods: SWT soft thresholding, SWT hard thresholding, ISSA-VMD, ISSA-VMD with SWT soft thresholding, and ISSA-VMD with SWT hard thresholding, on ECG signals with 5dB and 10dB noise. Ten models were established: 5dB-SWT-Soft, 5dB-SWT-Hard, 5dB-ISSA-VMD, 5dB-ISSA-VMD-SWT-Soft, 5dB-ISSA-VMD-SWT-Hard, 10dB-SWT-Soft, 10dB-SWT-Hard, 10dB-ISSA-VMD, 10dB-ISSA-VMD-SWT-Soft, and 10dB-ISSA-VMD-SWT-Hard. The evaluation metrics for each model are shown in Table 3.

**Table 3:** The results of two-factor analysis of variance

Source of Variation	DF	Sum of Squares	Mean Square	*F* Value	p Value
Denoising Method	3	9154.76	3051.59	2224.87	<0.0001
State of Fatigue	2	57.35	28.68	20.91	<0.0001
Interaction	6	20.00	3.33	2.43	0.0321
Error	378	518.46	1.37		
Total	383	9730.57			

When using the SWT soft and hard thresholding methods alone, the denoising performance is relatively poor, regardless of whether the noise level is 5 dB or 10 dB. The NCC is low, indicating low similarity between the reconstructed signal and the original signal. The MSE is high, indicating a large mean square error between the reconstructed signal and the original signal. The PE is also high, indicating a large peak error. This result may be because the SWT method may not handle signal details well when processing ECG signals, leading to a decrease in the quality of the reconstructed signal.

The denoising performance of the ISSA-VMD method alone is also not satisfactory. Although the NCC value is high, indicating a high similarity between the reconstructed signal and the original signal, the MSE value is still large, indicating a lower quality of the reconstructed signal. The PE value is also relatively high, indicating a large peak error. This suggests that the ISSA-VMD method may not effectively remove noise when processing ECG signals, leading to a decrease in the quality of the reconstructed signal. However, when combining ISSA-VMD with the second-generation wavelet soft and hard thresholding methods, namely the ISSA-VMD-SWT-Soft and ISSA-VMD-SWT-Hard models, the denoising performance improves. These models demonstrate better performance in terms of NCC, MSE, and PE metrics. The NCC value is relatively high, the MSE value is relatively low, and the PE value is relatively small, indicating a high similarity between the reconstructed signal and the original signal, good quality of the reconstructed signal, and small peak error. In summary, for denoising ECG signals, using the SWT soft and hard thresholding methods or the ISSA-VMD method alone yields unsatisfactory results. However, combining ISSA-VMD with the SWT soft and hard thresholding methods, especially with the soft thresholding, can achieve better denoising performance and improve the quality and similarity of the reconstructed signal. The evaluation metrics for each model are shown in Table 4.

**Table 4:** Denoise indicates value of the denoised ECG signal

Model	NCC	MSE	SNR	PE	Model	NCC	MSE	SNR	PE
5dB-SWT-Soft	0.368	0.584	11.606	0.340	10dB-SWT-Soft	0.374	0.784	19.775	0.922
5dB-SWT-Hard	0.374	0.687	10.820	0.353	10dB-SWT-Hard	0.368	0.776	19.606	0.995
5dB-ISSA-VMD	0.368	0.534	11.606	0.348	10dB-ISSA-VMD	0.388	0.484	20.752	0.822
5dB-ISSA-VMD-SWT-Soft	0.368	0.507	11.620	0.283	10dB-ISSA-VMD-SWT-Soft	0.374	0.716	22.626	0.795
5dB-ISSA-VMD-SWT-Hard	0.368	0.484	13.606	0.240	10dB-ISSA-VMD-SWT-Hard	0.375	0.484	22.975	0.722

In conclusion, for denoising sEMG signals, combining ISSA-VMD with the SWT and the hard thresholding method yields good results. For denoising ECG signals, ISSA-VMD combining with SWT the soft thresholding method achieves satisfactory results. It is important to note that these comparative results are based on experiments conducted with simulated noisy data. When applied to real signals, the effectiveness may be influenced by the characteristics of the actual signals and the types of noise present.

### Comparison of real sEMG and ECG noise reduction

**Figure 6:**
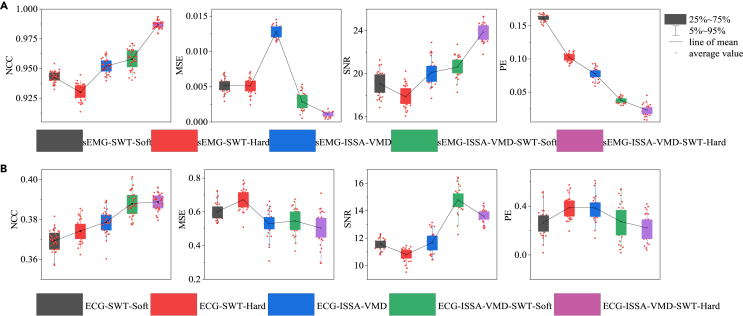
Real sEMG and ECG denoised index values (A) Real sEMG denoising. (B) Real ECG denoising. Different colors in the figure represent denoising methods respectively SWT-Soft, SWT-Hard, ISSA-VMD, ISSA-VMD-SWT-Soft, ISSA-VMD-SWT-Hard.

To validate the superiority of the ISSA-VMD and SWT algorithms in real sEMG and ECG signals, we collected sEMG and ECG signals from subjects and evaluated their performance using various metrics, as shown in Figure 6. Detailed parameters and standard deviations for each model are presented in Table S1. Concerning sEMG signal denoising, the method that combines ISSA-VMD with the SWT hard threshold technique outperforms other methods, exhibiting the highest NCC (0.988) and the lowest MSE (0.001), along with the highest SNR (24.060) and the lowest PE (0.020). It demonstrates excellent denoising performance, handling signal details better while effectively preserving useful signals. For ECG signal denoising, the method combining ISSA-VMD with SWT soft threshold technique shows better performance, with higher NCC (0.388) and lower MSE (0.527), along with higher SNR (14.622) and lower PE (0.273). These results align with denoising conclusions drawn from simulated noisy signals.

### Effect of ISSA-VMD-SWT on classification of body fatigue

#### Overall performance analysis

**Figure 7:**
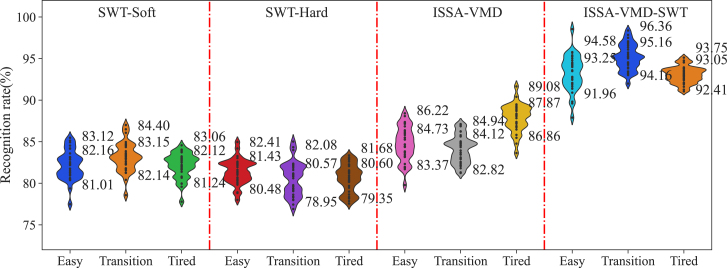
The recognition rate of sports fatigue of different models The values marked in the figure are the first quartile value, the average value and the third quartile value of the model respectively.

Based on the previous research, the workflow for fatigue classification was adjusted using the combined sEMG and ECG signals. For sEMG, The ISSA-VMD-SWT method is combined with hard threshold technology, while for ECG, the ISSA-VMD-SWT method is combined with the soft thresholding technique for denoising. The classification model established using these methods is named ISSA-VMD-SWT. To compare the performance, we also established three other models: SWT-Soft, SWT-Hard, and ISSA-VMD. The accuracy of these models is shown in Figure 7.

Based on the statistical results, the models are ranked in descending order of average accuracy as ISSA-VMD-SWT, ISSA-VMD, SWT-Soft, and SWT-Hard. The larger the distribution on the Y axis of the violin plot, the higher the variance in model accuracy, indicating significant variations in accuracy among individuals. From the perspective of variance, the ISSA-VMD-SWT model performs better, indicating that it can overcome individual differences. Comparing with previous research results (average accuracies of 98.75%, 92.25%, and 94.25% for Easy, Transition, and Tired, respectively), the accuracy of Transition recognition improved by 2.91% and the lowest accuracy improved by 4.16% after applying the denoising method proposed in this study. The range of recognition rates for the three states further narrowed, indicating improved adaptability of the model after denoising. However, the improvement was not significant, which may be due to the differences in the fatigue information used in the two experiments. The previous study mainly relied on self-assessment using the Rating of Perceived Exertion (RPE) scale, which may have had issues with unclear transition states. In contrast, this study employed the more scientific Ultima GX system.

Additionally, a comparison was made between the average accuracies of different genders. The average accuracy for males was 82.9% and for females was 82.91%. The two-factor ANOVA for gender and fatigue status and denoising methods are shown in Tables S2 and S3 No significant difference was found in the evaluation of exercise fatigue between genders, indicating that this denoising method is applicable to both male and female athletes.

#### Confusion matrix evaluation

**Figure 8:**
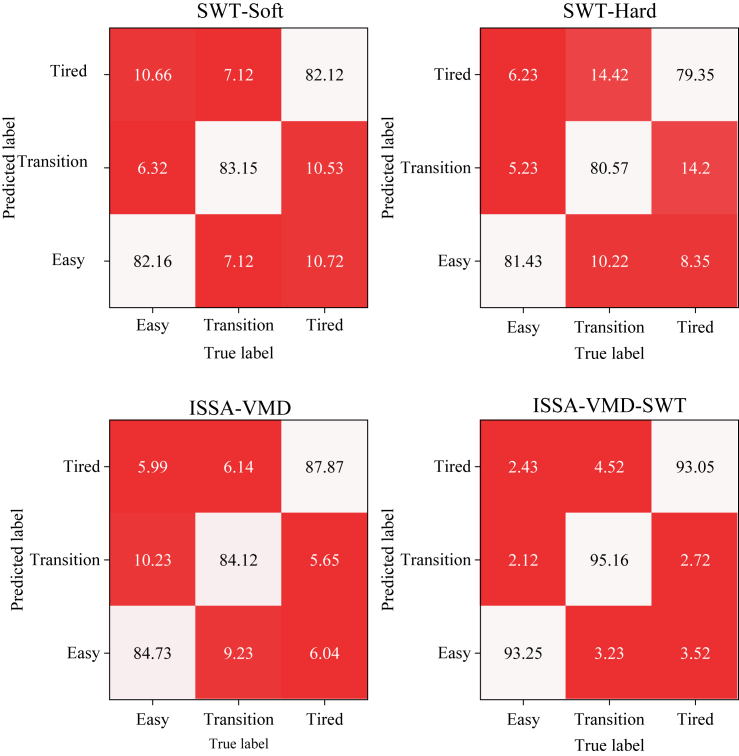
Confusion matrix accuracy evaluation of fatigue state classification models based on different denoising methods

Different models established by various denoising methods correspond to confusion matrices shown in Figure 8. Precision, Recall, and F1 Score were calculated based on statistical principles, and detailed results are in Table S4. Precision, Recall, and F1 Score are crucial metrics in assessing classification algorithm quality, especially considering F1 Score, which integrates Precision and Recall, serving as a comprehensive evaluation metric. Regarding the SWT-Soft model, it exhibits higher Precision and Recall in the Easy category, indicating its strong predictive ability for this class. However, Precision and Recall are slightly lower for the Transition and Tired categories. SWT-Hard generally demonstrates slightly lower Precision, Recall, and F1 Score in the Easy, Transition, and Tired categories compared to SWT-Soft, particularly evident in the Transition and Tired categories. This suggests potential errors or biases in SWT-Hard when predicting these categories, resulting in slightly inferior performance compared to SWT-Soft. The ISSA-VMD-SWT model showcases relatively higher Precision, Recall, and F1 Score across the Easy, Transition, and Tired categories. These results indicate that these models perform well across multiple categories, especially achieving a high Precision level in the Easy category, signifying high predictive accuracy in this class. These outcomes highlight performance disparities among different models across various categories, indicating that certain models might excel in specific fatigue types. Further exploration might be necessary to understand the reasons for differences among fatigue states.

#### Variance analysis

Conducting two-way ANOVA on the data, where the first factor is set as the denoising method and the second factor as the fatigue state. The interaction plot for the two factors is illustrated as Figure S1. The results of two-factor ANOVA are shown in Table 3. At a significance level of p < 0.05, there exists a significant difference in the overall means between the Denoising Method and State of Fatigue. The interaction between these two factors has been investigated. Statistical analysis of differences among various groups has been carried out to deeply comprehend the impact of different factors on the study outcomes.

The denoising method significantly impacts the recognition rate (F(3,378)=2224.87, p<0.0001). Simultaneously, the fatigue state also significantly influences the recognition rate (F(2,378)=20.91, p<0.0001). There is an interaction effect between these two factors (F(6,378)=2.43, p=0.0321). Among them, 'ISSA-VMD-SWT' exhibits a relatively higher recognition rate. The Tired state exhibits a higher recognition rate compared to "Easy" and "Transition"; this might be attributed to a sudden increase in oxygen consumption and significant muscle contractions. Figure 1 vividly displays the differences in the Tired state. Follow-up post-hoc analyses were conducted, and the results are shown in Tables S5 and S6. The q values and probabilities in the tables indicate significant differences between various denoising methods and fatigue states in many cases. Confidence interval data display considerable differences among different denoising methods in the Transition state, while the variation in confidence intervals is smaller in the Tired state. Based on the grouping description of the interaction, it can be concluded that ISSA-VMD-SWT has relatively minor statistical differences in the Transition, Easy, and Tired states. In essence, ISSA-VMD-SWT can effectively overcome muscle conditions under different fatigue states, demonstrating stronger generalization capabilities.

### ISSA-VMD-SWT compared with other methods

Select two more advanced methods of human weak current signal denoising, Combines the traditional wavelet threshold denoising with wavelet digital filter threshold denoising (WDFTD) and empirical wavelet transform (EWT) and improved interval thresholding (EWT-IIT).^14^ Based on the fusion sEMG and ECG experimental data and ISSA-VMD-SWT compared processing signal parameters, as well as the recognition accuracy of muscle fatigue state. The differences among the three algorithms are shown in Table 5. NCC and MSE indicators show that the ISA-VMD-SWT algorithm performs well in preserving the original signal characteristics. SNR and PE display success This method successfully reduces signal noise and has little change in signal amplitude. Table S2 shows the difference in Precision, Recall and F1 Score caused by different denoising methods. Compared with the three algorithms, ISA-VMD-SWT has certain advantages in different parameters of the three states.

**Table 5:** Comparison of denoising effects of various algorithms

Model	NCC	SD	MSE	SD	SNR	SD	PE	SD
WDFTD	0.63529	0.21202	0.00492	0.003	19.091	3.441	0.498	0.199
EWT-IIT	0.6529	0.24248	0.005	0.003	21.527	2.738	0.442	0.189
ISSA-VMD-SWT	0.79571	0.22985	0.004	0.002	22.811	2.773	0.270	0.192

### Conclusion

This study introduces a novel fatigue recognition model by fusing sEMG and ECG signals to address the robustness against noises interfering weak electrical signals. The innovative method integrates ISSA-VMD and SWT denoising techniques. The optimization speed is accelerated by introducing the Tent chaotic map. The soft or hard threshold of SWT can effectively retain the fatigue-related features and reduce the noise level. The comprehensive evaluations involving both simulated noisy signals and real-world data demonstrate substantial advantages. Different from the GA, PSO, and SSA, the ISSA exhibits a faster convergence rate and lower fitness value. The searching mechanism of the ISSA-VMD model makes it easy to escape from local optima. The combination of ISSA-VMD with SWT soft or hard threshold achieves superior values in terms of NCC, MSE, SNR, and PE. The ISSA-VMD-SWT obtains significantly higher recognition rates for three fatigue levels. These results indicate that the proposed model possesses higher recognition accuracy and better generalization performance. Specifically, the recognition rates for Easy, Transition, and Tired levels are 93.25%, 95.16%, and 93.05%, respectively. The reduced inter-subject variance of fatigue levels highlights the enhanced generalization ability of the model. Furthermore, gender shows slight influence on fatigue classification, indicating that there is no significant difference in fatigue estimation between males and females.

In conclusion, the signal denoising method based on ISSA-VMD-SWT soft and hard threshold exhibits superior performance in muscle fatigue recognition models using sEMG and ECG signals. This method holds great potential for practical applications in the fields of exercise physiology, engineering, and rehabilitation medicine. Future research of our team will focus on improving feature extraction and classification models for weak biological electrical signals. The eventual goal is to integrate the entire technology, involving signal acquisition, processing, and recognition, into embedded devices such as portable wearable devices. This will facilitate the advancement of sports science toward informatization.

### Limitations of the study

Despite the promising results, this study has several limitations. First, the method’s effectiveness is primarily validated in controlled environments with a limited number of participants, which may not fully represent the diverse conditions encountered in real-world applications. Second, while the ISSA-VMD-SWT approach shows significant improvements in noise reduction and fatigue recognition, its computational complexity is higher compared to simpler models, which may impact its real-time application in wearable devices. Third, the study focuses on the general population without considering specific groups that may have different physiological responses, such as athletes or individuals with health conditions. Lastly, the influence of gender on fatigue classification, although slight, suggests potential variability in the model’s performance across different demographic groups, which was not extensively explored.

Future studies will focus on broadening the model’s applicability through diversifying participant demographics, enhancing computational efficiency for real-time applications, tailoring models for specific populations, conducting in-depth demographic variability analysis, integrating technology into wearable devices, and refining feature extraction and classification techniques. These efforts aim to solidify the model’s utility in exercise physiology, rehabilitation, and healthcare monitoring, pushing forward the practical implementation of advanced fatigue recognition in wearable technology.

## STAR★Methods

### Key resources table

**Table:** 

REAGENT or RESOURCE	SOURCE	IDENTIFIER
**Biological samples**		

a total of 18 male participants and 14 female participants	School of Sports Medicine and Rehabilitation, North Sichuan Medical College	

**Software and algorithms**		

Python(version 3.7.15)	Python Software Foundation	https://www.python.org/
Pandas(version`.2.4)	Python package	https://pandas.pydata.org/
scikit-learn(version 1.0.2)	Python package	https://scikit-learn.org/stable/
Numpy(version 1.26)	Python package	https://numpy.org/
OriginPro 2024(10.1.0.178)	Originlab	https://www.originlab.com/

### Resource availability

#### Lead contact

Further information and requests for resources related to this study should be directed to, and will be fulfilled by, the lead contact Hao Li (jacky809@nsmc.edu.cn).

#### Materials availability

This study did not generate new unique reagents.

#### Data and code availability

This paper does not report original code.
Any additional information required to reanalyze the data reported in this paper is available from the lead contact upon request.

### Experimental model and study participant details

#### Data collection

This research project was conducted by the North Sichuan Medical College in accordance with the ethical code of the World Medical Association. Recruiting volunteers began in May 2022 and ended in July 2022. The study was approved by the Ethics Committee of the North Sichuan Medical College (No. 2020ER(R)017). All respondents signed the consent forms prior to taking measurements and participating in the interview sessions. To ensure the scientific validity and reliability of the experiment, a total of 18 male participants and 14 female participants were selected, aged between 19 and 24 years old, with heights ranging from 160.5 cm to 182.0 cm and weights ranging from 60.12 kg to 77.50 kg.

All participants were in good health and had no history of cardiovascular diseases or severe muscle injuries. They refrained from consuming caffeine, nicotine, and alcohol within 24 h prior to the experiment, as well as from engaging in vigorous exercise. All participants were adequately informed about the specific details, objectives, methods, and potential risks of the study, and they volunteered to participate in the experiment. This study strictly adhered to the principles of the Helsinki Declaration and complied with Chinese laws and regulations. The experiment will be conducted from January to May 2023.

To ensure accurate power regulation, the exercise tests were conducted using the Lode Corival CPET cycle ergometer. Ag-AgCl surface electrodes were arranged in a bipolar configuration (center-to-center distance of 20 mm, diameter of 1 cm), and the Noraxon Ultium sEMG sensor was used to record sEMG signals during the experiment. The Noraxon Ultium sEMG sensor had a sampling frequency of 2000 Hz. Noraxon MR3 software was used for offline analysis of the collected sEMG signals. The ECG equipment used was the Philips PageWriter TC50, a 12-lead ECG machine from Philips Healthcare in the Netherlands. The Ultima GX system was used to measure participants ventilation volume (VE), oxygen uptake (${\text{VO}}_{2}$), and carbon dioxide production (${\text{VCO}}_{2}$) during the exercise test. All sensors and experimental equipment were calibrated prior to the experiment.^15^

It was necessary to shave off excess hair on the participants' leg skin and wipe it with medical alcohol to reduce impedance before the experiment. Following the SENIAM (sEMG for Non-Invasive Assessment of Muscles)^16^ guidelines, we placed the sEMG sensors at the appropriate positions of the rectus femoris (RF), vastus lateralis (VL), vastus medialis (VM), and gastrocnemius (GA) muscles. To ensure that participants were not affected during the procedure, the sEMG sensors were secured using athletic tape. ECG sensors were positioned on both sides of the fourth, fifth, and sixth intercostal spaces of the participants' chest and along the midline below the heart margin. CH represents the ECG sensors, while RF, VL, VM, and GA represent the corresponding positions of the sEMG sensors. Prior to testing, participants familiarized themselves with the equipment and procedures. After a 3-min warm-up on a cycle ergometer, each participant performed incremental exercise at room temperature (26 ± 3°C), starting at an initial workload of 100 W and increasing by 25 W/m. Participants maintained a cycling speed of 70–75 r/m throughout the entire testing process. The test was terminated when participants were unable to maintain 70 r/m. Each participant underwent the same test for three consecutive days, with a 24-h interval between tests. After the experiment, we analyzed the slope of the ${\text{VCO}}_{2}$ and ${\text{VO}}_{2}$ kinetics curves^17^ and calculated the anaerobic threshold (AT) using the V-slope method. The sEMG and ECG corresponding to the time of AT were categorized as "Easy," "Transition," and "Tired."

#### Signal preprocessing

The original sEMG and ECG signal contains noise interference, which needs to be preprocessed. Firstly, the original ECG and sEMG signals were filtered by 0∼100 Hz and 0–500 Hz low-pass filters to remove high-frequency interference. Secondly, 49.5–50 Hz adaptive notch filters were used to filter the power frequency and harmonic interference in the signal. Finally, empirical mode decomposition(EMD) and discrete wavelet transform(DWT) domains were used to reduce the noise11. which reduce the noise from the initial IMFs instead of discarding them completely thus yielding a relatively cleaner ECG signal.

#### Noise reduction algorithm based on ISSA-VMD-SWT

##### Variational mode decomposition (VMD)

VMD as a method with the characteristics of Wiener filtering and adaptive signal decomposition, effectively addresses the issues of mode mixing and high-frequency signal loss. It performs well in handling nonlinear signals.^18^ The specific decomposition process of the signal is as follows.

(1) The initialization of parameters in VMD is based on the optimization model and constraints of the variational problem. The optimization model and constraints for the variational problem are as follows:

(Equation 1) $$\left\{ \begin{array}{c} \overset{\min}{\left\{u_{k}\right\}\left\{w_{k}\right\}}\sum_{k}{\left\|\partial_{t}\left[\left(\delta \left(t\right)+\frac{j}{\pi t}\times u_{k}\left(t\right)\right)\right]e^{-jw_{k}t}\right\|}^{2}\\ \sum_{k}u_{k}\left(t\right)=f\left(t\right) \end{array}\right.$$

where $u_{k}\left(t\right)$ represents the k-th mode component obtained from the decomposition of the original signal f(t), $w_{k}$ represents the central frequency of each component.

(2) By introducing an augmented Lagrange function, the constrained variational problem can be transformed into an unconstrained variational problem as follows:

(Equation 2) $$L\left(\left\{u_{k}\right\},\left\{w_{k}\right\},\lambda \right)=\alpha \sum_{k=1}^{K}{\left\|\partial_{t}\left[\left(\delta \left(t\right)+\frac{j}{\pi t}\times u_{k}\left(t\right)\right)\right]e^{-jw_{k}t}\right\|}^{2}+{\left\|f\left(t\right)-\sum_{w_{k}}^{K}u_{k}\left(t\right)\right\|}^{2}+\left\langle \lambda \left(t\right),f\left(t\right)-\sum_{w_{k}}^{K}u_{k}\left(t\right)\right\rangle$$

(3) The augmented Lagrange function in Equation 2 can be solved using the alternating direction method of multipliers (ADMM). This iterative algorithm aims to find the optimal solution of the model by updating the mode components and Lagrange multipliers alternately. The steps of the ADMM algorithm for solving Equation 2 can be outlined as follows:

(Equation 3) $$u_{k}^{n+1}\left(w\right)=\frac{\hat{f}\left(w\right)-\sum_{i\neq k}{\hat{u}}_{i}\left(w\right)+\hat{\lambda }\left(w\right)/2}{1+2\alpha {\left(w-w_{k}\right)}^{2}}$$

(Equation 4) $$w_{k}^{n+1}=\frac{\int {\left|{\hat{u}}_{k}\left(w\right)\right|}^{2}dw}{\int_{0}^{\infty }{\left|{\hat{u}}_{k}\left(w\right)\right|}^{2}dw}$$

Where $u_{k}^{n+1}\left(w\right)$ and $w_{k}^{n+1}$ are the wiener filter and frequency center of each component. It can be seen from the VMD decomposition process that the empirical selection of parameters k and α in practical applications may not achieve the best decomposition performance or even appear mode aliasing. Therefore, how to self-apply the selection of VMD parameters is the key to accurate decomposition.

##### Improved sparrow search algorithm (ISSA)

In order to achieve better decomposition and denoising effects, this study utilizes intelligent optimization algorithms to optimize the key parameters, k and α, of VMD within a reasonable range. The SSA is derived from the foraging and anti-predation processes of sparrows, possesses strong global optimization ability and local search capability.^19^ However, it suffers from issues such as uneven initial population distribution, slow convergence speed, and susceptibility to local optima in later stages.^20^ In this study, an improved Tent chaotic mapping is introduced to optimize the initial population, thereby improving the optimization speed. Simultaneously, the number of discoverers, joiners, and scouts is optimized to enhance the global search ability in the early stages and the search capability in the later stages, thus avoiding being trapped in local optima. The specific steps are as follows:

(1) Based on the improved Tent chaotic mapping, a sequence $y_{i}$ is generated, and this sequence is mapped to different individual sparrows to improve the population distribution.

(Equation 5) $$\left\{ \begin{array}{cc} y_{i+1}=2y_{i}+\frac{\text{rand}\left(0,1\right)}{N\times T_{\max}}\text{,} & y_{i}\leq 0.5\\ y_{i+1}=2\left(1-y_{i}\right)+\frac{\text{rand}\left(0,1\right)}{N\times T_{\max}}\text{,} & y_{i}>0.5 \end{array}\right.$$

(Equation 6) $$\left\{ \begin{array}{c} X_{i}=b_{\min}+\left(b_{\max}-b_{\min}\right)\times y_{i}\\ X_{i}\in \left(b_{\min},b_{\max}\right) \end{array}\right.$$

Where $y_{i}$ represents the chaotic sequence; i=1,2,...,N; $y_{i}$ is a random number between 0 and 1. N represents the population size, $T_{\max}$ is the maximum number of iterations; $X_{i}$ represents the initial population; $b_{\min}$ and $b_{\max}$ denote the lower and upper limits of the search space.

(2) Adaptive weights are introduced to update the finder position as:

(Equation 7) $$\begin{array}{c} X_{i}^{n+1}=\left\{ \begin{array}{cc} X_{i}^{n}+\cos\left(\frac{\pi n}{2\times T_{\max}}\right)\times \gamma_{1}\times \left(X_{i}^{n}-X_{best}^{n}\right)， & R_{2}<ST\\ X_{i}^{n}+\gamma_{2}\times L， & R_{2}\geq ST \end{array}\right. \end{array}$$

Where $\begin{array}{c} X_{i}^{n} \end{array}$ is the position of the i sparrow of the N-th generation, $X_{best}^{n}$ is the best position for individual sparrows, n indicates the number of iterations, $\gamma_{1}$ and $\gamma_{2}$ are random numbers of (0,1) and random numbers that follow a normal distribution, respectively, L is a row of multidimensional all-one matrix, $R_{2}$ is an alarm value [0,1]. ST is a security threshold [0.5,1]. When $R_{2}＜ST$, the discoverer continued to search for food *in situ*, guiding the population to get better fitness, When $R_{2}\geq ST$, the finder should immediately move to a safe area to prevent predators.

(3) During the foraging process, if the discoverer finds a better food source, the joiner will immediately move toward it, and its position is updated as follows:

(Equation 8) $$X_{i}^{n+1}=\left\{ \begin{array}{cc} \beta_{2}\times \exp\left(\frac{X_{\text{worst}\;}^{n}-X_{i}^{n}}{i^{2}}\right), & i>\frac{N}{2}\\ X_{\text{best}\;}^{n}+\left|X_{i}^{n}-X_{\text{best}\;}^{n}\right|\times L\times A^{+}, & i\geq \frac{N}{2} \end{array}\right.$$

Where $X_{worst}^{n}$ is the worst position for individual sparrows, When i＞N/2, the i-th entrant will leave the original feeding ground and move to other food sources. When i≤N/2, the i-th entrant stays in place and forages.

(4) During the foraging process, when danger is detected, the warner will leave its current location and move toward safer food sources or approach other sparrows. The position of the warner is updated as follows:

(Equation 9) $$X_{i}^{n+1}=\left\{ \begin{array}{cc} X_{best}^{n}+\gamma_{3}\times \left|X_{i}^{n}-X_{bot}^{n}\right|, & C_{i}\neq C_{g}\\ X_{i}^{n}+\gamma_{4}\times \left(\frac{X_{i}^{n}-X_{best}^{n}}{\left(C_{i}-C_{w}\right)+\varepsilon }\right), & C_{i}=C_{g} \end{array}\right.$$

Where $\gamma_{3}$ and $\gamma_{4}$ represent random numbers following a normal distribution and [-1,1] uniform distribution. ε is a very small value, typically on the order of $10^{-50}$. $C_{i}$ is the fitness value of the current sparrow individual, and $C_{w}$ represents the fitness value of the global worst individual.

To improve the algorithm’s initial global search capability and avoid falling into local optima later on, the optimization of the number of explorers and sentinels is implemented. The numbers of explorers and sentinels are dynamically adjusted during the algorithm’s execution.

(Equation 10) $$pNum\;\left(t+1\right)=pNum\left(t\right)\times \left(e^{-\frac{t}{T_{\max}}}+1\right)$$

(Equation 11) $$yNum=S_{\max}-\mathrm{int}\left[{\left(\frac{t}{T_{\max}}\right)}^{2}\times \left(S_{\max}-S_{\min}\right)\right]$$

Where pNum and yNum are the numbers of explorers and sentinels. The initial number of explorers, pNum(1)=0.3. $S_{\max}$ and $S_{\min}$ are the maximum and minimum numbers of sentinels, which are defined as 0.2× N and 0.05× N.

##### Fitness function

Selecting an appropriate fitness function is crucial for the improvement of the SSA optimization. Based on the optimal theory of VMD, it is known that when the number of modes k and the penalty factor α are properly chosen, the sum of energies of the decomposed modes equals the energy of the original signal. The residual mode exhibits a low correlation with the original spectrum. In this study, the Quality Factor $Q_{f}$ is selected as the measure of the decomposition performance. A smaller value of $Q_{f}$ indicates a better decomposition result. By considering the distinguish ability of the modes, the occurrence of mode mixing and spurious components is further avoided.

(Equation 12) $$Q_{f}=\frac{\left(E_{x_{i}}-\sum_{i=1}^{k}E_{VMF_{i}}\right)\times r}{\sum_{i=1}^{k}{\left(H_{i}-\bar{H}\right)}^{2}}$$

Where $E_{x_{i}}$ is energy value of the original signal, $E_{VMF_{i}}$ is the energy value of the VMF component, r is the correlation coefficient between the residual component of VMD decomposition and the original signal, $H_{i}$ is the information entropy of each VMF component, $\bar{H}$ is the average of the information entropy of k VMFS

##### The second generation wavelet denoising

The second-generation wavelet transform (SWT) is a relatively new wavelet theory that has emerged in recent years. Compared to traditional wavelets, SWT construction avoids the use of Fourier transforms, resulting in faster computational speed. The transformation process of SWT includes two main steps: decomposition and reconstruction. The decomposition process can be summarized as follows.

(1) Decomposition, raw signal X={x[n],n∈Z} is decomposed into two disjoint subsets, odd sample set $X_{0}=\left\{x_{0}\left[0\right],n\in Z\right\}$ and even sample set $X_{e}=\left\{x_{e}\left[n\right],n\in Z\right\}$.

(Equation 13) $$x_{0}\left[n\right]=x\left[2n+1\right]$$

(Equation 14) $$x_{e}\left[n\right]=x\left[2n\right]$$

The better the correlation between $X_{0}$ and $X_{e}$, the better the decomposition result. This decomposition method utilizes the local correlation of the signal, providing a data foundation for subsequent prediction and updating processes.

(2) Prediction is performed using the correlation between data by employing a prediction operator P, which is independent of the dataset, to predict the even sample set $X_{e}$ based on the odd sample set $X_{0}$. The error d[n] between $x_{0}\left[n\right]$ and $P\left(X_{e}\right)$ is expressed as follows:

(Equation 15) $$d\left[n\right]=x_{0}\left[n\right]-P\left(X_{e}\right)$$

(3) The updata operator U is used to updata the detail coefficient D={d[n],n∈Z}, add U(D) to $x_{e}\left[n\right]$, and calculate the approximation coefficient c[n] as:

(Equation 16) $$c\left[n\right]=x_{e}\left[n\right]+U\left(D\right)$$

After the above three steps, the detail coefficient D={d[n],n∈Z} and approximation coefficient c[n] is obtained. Multilayer decomposition of SWT can be realized through several iterations. The prediction P=[p(1),p(2),⋯,p(M)] and updata operators U=[u(1),u(2),⋯,u(N)] are vectors of length M and N. The above three steps constitute a complete ascension process. The reconstruction process of the second generation wavelet transform is to execute the decomposition process in reverse order and transform the positive and negative signs simultaneously.

The main objective of SWT denoising is to set thresholds for the wavelet coefficients generated during the decomposition process and process them accordingly. Subsequently, the processed wavelet coefficients are inverse transformed to obtain the denoised signal. Soft and hard thresholding methods are commonly used for threshold processing. The calculation methods for soft and hard thresholds have been reported multiple times in previous studies. However, both soft and hard thresholding methods have certain limitations. The signal reconstructed using the hard thresholding method does not preserve the smoothness of the original signal, while the signal reconstructed using the soft thresholding method may introduce some bias. Therefore, to achieve effective denoising, the SWT denoising with soft and hard thresholding methods needs to be combined with other approaches.

##### Noise reduction process

To overcome the limitations of empirical parameter setting in VMD and eliminate noise from the signals, this study employs the ISSA to optimize the key parameters k and α in VMD. By integrating the advantages of VMD decomposition and the SWT, a denoising method for sEMG and ECG signals based on ISSA-VMD and the SWT is proposed. The specific steps are as follows:

(1) Different states of human sEMG signals were collected using an electrophysiological signal acquisition device. The signals were then subjected to preprocessing steps such as bandpass filtering and smoothing filtering.
(2) The processed signal xi was subjected to ISSA-VMD decomposition, resulting in k VMF components.
(3) The spectral correlation coefficient of each VMF component was calculated, and components with a correlation coefficient below 0.1 were discarded as spurious components, resulting in the identification of valid components.
(4) The valid components obtained from the previous step were subjected to denoising and reconstruction using the SWT, resulting in a denoised signal.

##### Noise reduction evaluation index

In order to objectively compare the performance of various denoising algorithms, this study selected normalized correlation coefficient (NCC), mean square error (MSE), signal-to-noise ratio (SNR), and peak error (PE) as evaluation metrics for denoising performance. A higher value of SNR indicates less noise in the reconstructed signal, while a larger waveform similarity coefficient indicates a closer resemblance between the reconstructed signal and the original signal.

NCC is defined as the degree of distortion of the reconstructed signal relative to the original signal waveform. The calculation formula is as follows:

(Equation 17) $$NCC=\frac{\sum_{i=1}^{N}x_{i}^{\prime }\times x_{i}}{\sqrt{\left(\sum_{i=1}^{N}{\left(x_{i}^{\prime }\right)}^{2}\right)\left(\sum_{i=1}^{N}x_{i}^{2}\right)}}$$

MSE is defined as the average of the squared differences between the original signal and the reconstructed signal. The calculation formula is as follows:

(Equation 18) $$MSE=\frac{1}{N}\sum_{i=1}^{N}{\left(x_{i}-x_{i}^{\prime }\right)}^{2}$$

SNR is defined as the ratio of the power of the original signal to the power of the noise signal. The calculation formula is as follows:

(Equation 19) $$SNR=10\;\log_{10}\left(\frac{\sum_{i=1}^{N}x_{i}^{2}}{\sum_{i=1}^{N}{\left(x_{i}-x_{i}^{\prime }\right)}^{2}}\right)$$

PE is defined as the maximum difference between the original signal and the reconstructed signal. The calculation formula is as follows:

(Equation 20) $$PE=\max_{i=1}^{N}\left(\left|x_{i}-x_{i}^{\prime }\right|\right)$$

Where $x_{i}$ represents the original signal, and $x_{i}^{\prime }$ is the reconstructed signal. N indicates the data length. Based on the above three evaluation indicators, the advantages and disadvantages of each method are compared.

#### Establishment of body fatigue recognition model after sEMG and ECG denoising

Comparing the accuracy of fatigue recognition in sEMG and ECG signals using different denoising methods to evaluate the improvement of the proposed denoising method on the assessment of muscular weak electrical signal fatigue. To establish evaluation metrics, we will adopt recognition rate as the measurement criterion. The recognition rate refers to the ratio of correctly identified samples to the total number of samples. Through preprocessing and denoising, our aim is to improve the quality and accuracy of the signals, thereby enhancing the ability of fatigue recognition. Referring to our previous research,^20^ we will use a feature fusion model based on Support Vector Machine (SVM) to perform fatigue recognition by combining preprocessed and denoised ECG and sEMG signals. By comparing the experimental results using different denoising methods, we can evaluate the impact of each method on the recognition rate. A higher recognition rate indicates a better improvement in the assessment of muscular weak electrical signal fatigue using the denoising method, enabling more accurate identification of fatigue states. At the same time, we also need to consider factors such as algorithm complexity, real-time performance, and applicability to different signal types to comprehensively evaluate the advantages and disadvantages of various methods and select the most suitable method for muscular weak electrical signal fatigue assessment tasks. The formula for calculating the recognition rate is as follows:

(Equation 21) $$\text{Recognition}\;\text{rate}=\frac{\text{number}\;\text{of}\;\text{samples}\;\text{correctly}\;\text{identified}\;}{\text{total}\;\text{number}\;\text{of}\;\text{test}\;\text{samples}}\times 100\%$$

### Quantification and statistical analysis

Statistical analyses were performed using custom python. Details of all statistical analyses can be found above in the relevant subsections of the method details section. Sample number (n) and statistical methods used to assess differences between groups are indicated in the relevant subsections of the result section.
