# Supplementary material for: Research on the recognition model of exercise fatigue based on the fusion of sEMG and ECG signals
Source: iScience. 2024 Feb 29;27(4):109365. doi: 10.1016/j.isci.2024.109365 (PMC10951635; doi:10.1016/j.isci.2024.109365)
Supplement: Document S1. Figure S1 and Tables S1–S6 [file mmc1.pdf]

iScience, Volume *27*

Supplemental information

Research on the recognition model of exercise fatigue based on the fusion of sEMG and ECG signals

Hao Li and Dujuan Li

Suppl. Material

Suppl. Table

Table S 1 Real sEMG and ECG signal denoising effect parameter table, related to Figure 1.

Model	NCC	SD	MSE	SD	SNR	SD	PE	SD
sEMG-SWT-Soft	0.942	0.004	0.005	9.42E-4	19.201	1.086	0.161	0.004
sEMG-SWT-Hard	0.929	0.006	0.005	0.001	18.125	0.991	0.101	0.005
sEMG-ISSA-VMD	0.952	0.006	0.013	8.64E-4	19.967	1.154	0.078	0.008
sEMG-ISSA-VMD-SWT-Soft	0.957	0.008	0.003	0.001	20.594	0.925	0.038	0.004
sEMG-ISSA-VMD-SWT-Hard	0.986	0.003	0.001	3.44E-4	24.060	0.787	0.020	0.007
ECG-SWT-Soft	0.369	0.005	0.598	0.052	11.606	0.391	0.351	0.112
ECG-SWT-Hard	0.374	0.005	0.687	0.059	10.920	0.467	0.363	0.088
ECG-ISSA-VMD	0.378	0.006	0.534	0.074	11.646	0.756	0.368	0.101
ECG-ISSA-VMD-SWT-Soft	0.388	0.006	0.527	0.073	14.622	0.908	0.273	0.145
ECG-ISSA-VMD-SWT-Hard	0.389	0.004	0.494	0.092	13.644	0.465	0.250	0.103

Table S 2 Two-factor analysis of population variance between gender and fatigue status

	DF	Sum of squares	Mean square	F value	P value
Gender	1	1.04363	1.04363	2.99878	0.08675
Fatigue state	2	24.29102	12.14551	34.89891	<0.0001
interaction	2	0.16729	0.08365	0.24035	0.78685
Model	5	25.92979	5.18596	14.90133	<0.0001
Error	90	31.32178	0.34802		
Modified whole	95	57.25156			

*At the 0.05 level, the overall mean for Gender is not significantly different. The overall mean Fatigue state is significantly different. The correlation between Gender and Fatigue state was not significant

Table S 3 Two-factor analysis of population variance between gender and Denoising Method

	DF	Sum of squares	Mean square	F value	P value
Gender	1	0.00592	0.00592	0.0133	0.9084
Denoising Method	3	2826.30308	942.10103	2116.57489	<0.0001
interaction	3	1.58587	0.52862	1.18763	0.31746
Model	7	2872.66884	410.38126	921.98464	<0.0001
Error	120	53.41277	0.44511		
Modified whole	127	2926.08161			

*At the 0.05 level, the overall mean for Gender is not significantly different. The Denoising Method is significantly different. The correlation between Gender and Denoising Method is not obvious.

Table S 4 The Precision, Recall and F1 Score of the model established by different denoising methods , related Figure 3.

SWT-Soft	Precision	Recall	F1 Score	ISSA-VMD	Precision	Recall	F1 Score
Easy	82.83%	82.16%	82.5%	Easy	82.12%	84.73%	83.41%
Transition	85.36%	86.09%	85.72%	Transition	83.78%	84.12%	83.95%
Tired	79.43%	82.22%	80.8%	Tired	87.64%	87.87%	87.75%
**SWT-Hard**	**Precision**	**Recall**	**F1 Score**	**ISSA-VMD-SWT**	**Precision**	**Recall**	**F1 Score**
Easy	78.69%	81.43%	80.04%	Easy	92.98%	93.25%	93.11%
Transition	78.29%	80.57%	79.42%	Transition	94.88%	95.16%	95.02%
Tired	80.98%	79.35%	80.16%	Tired	93.78%	93.05%	93.41%
**WDFTD**	**Precision**	**Recall**	**F1 Score**	**EWT-IIT**	**Precision**	**Recall**	**F1 Score**
Easy	83.12%	83.73%	83.42%	Easy	79.69%	80.43%	80.06%
Transition	83.68%	82.12%	82.89%	Transition	75.39%	82.57%	78.82%
Tired	84.24%	86.87%	85.53%	Tired	81.98%	81.35%	81.66%

Table S 5 Results of Turkey's HSD interaction analysis, related Figure 1.

Denoising Method	State of fatigue	Denoising Method	State of fatigue	Mean difference	*q* value	Prob	Sig	Lower bound of confidence interval	Upper limit of confidence interval
SWT-Soft	Transition	SWT-Soft	Easy	11.06	11.06	<0.0001	1	0.72	1.76
SWT-Soft	Tired	SWT-Soft	Easy	6.02	6.02	0.00153	1	0.15	1.20
SWT-Soft	Tired	SWT-Soft	Transition	5.04	5.04	0.02087	1	-1.09	-0.04
SWT-Hard	Easy	SWT-Soft	Easy	5.85	5.85	0.00255	1	-1.18	-0.13
SWT-Hard	Easy	SWT-Soft	Transition	16.91	16.91	<0.0001	1	-2.42	-1.38
SWT-Hard	Easy	SWT-Soft	Tired	11.87	11.87	<0.0001	1	-1.85	-0.81
SWT-Hard	Transition	SWT-Soft	Easy	1.19	1.19	0.99951	0	-0.66	0.39
SWT-Hard	Transition	SWT-Soft	Transition	12.26	12.26	<0.0001	1	-1.90	-0.85
SWT-Hard	Transition	SWT-Soft	Tired	7.22	7.22	<0.0001	1	-1.33	-0.29
SWT-Hard	Transition	SWT-Hard	Easy	4.65	4.65	0.04975	1	0.00	1.04
SWT-Hard	Tired	SWT-Soft	Easy	14.09	14.09	<0.0001	1	-2.10	-1.06
SWT-Hard	Tired	SWT-Soft	Transition	25.16	25.16	<0.0001	1	-3.34	-2.30
SWT-Hard	Tired	SWT-Soft	Tired	20.12	20.12	<0.0001	1	-2.78	-1.74
SWT-Hard	Tired	SWT-Hard	Easy	8.25	8.25	<0.0001	1	-1.45	-0.40
SWT-Hard	Tired	SWT-Hard	Transition	12.90	12.90	<0.0001	1	-1.97	-0.93
ISSA-VMD	Easy	SWT-Soft	Easy	23.86	23.86	<0.0001	1	2.15	3.20
ISSA-VMD	Easy	SWT-Soft	Transition	12.80	12.80	<0.0001	1	0.91	1.96
ISSA-VMD	Easy	SWT-Soft	Tired	17.84	17.84	<0.0001	1	1.48	2.52
ISSA-VMD	Easy	SWT-Hard	Easy	29.71	29.71	<0.0001	1	2.81	3.85
ISSA-VMD	Easy	SWT-Hard	Transition	25.05	25.05	<0.0001	1	2.29	3.33
ISSA-VMD	Easy	SWT-Hard	Tired	37.95	37.95	<0.0001	1	3.74	4.78
ISSA-VMD	Transition	SWT-Soft	Easy	21.68	21.68	<0.0001	1	1.91	2.95
ISSA-VMD	Transition	SWT-Soft	Transition	10.62	10.62	<0.0001	1	0.67	1.71
ISSA-VMD	Transition	SWT-Soft	Tired	1.24	15.66	<0.0001	1	1.23	2.28
ISSA-VMD	Transition	SWT-Hard	Easy	0.67	27.53	<0.0001	1	2.57	3.61
ISSA-VMD	Transition	SWT-Hard	Transition	-0.56	22.88	<0.0001	1	2.04	3.09
ISSA-VMD	Transition	SWT-Hard	Tired	-0.65	35.78	<0.0001	1	3.49	4.54
ISSA-VMD	Transition	ISSA-VMD	Easy	-1.89	2.17	0.92883	0	-0.77	0.28
ISSA-VMD	Tired	SWT-Soft	Easy	-1.33	53.59	<0.0001	1	5.49	6.53
ISSA-VMD	Tired	SWT-Soft	Transition	-0.13	42.53	<0.0001	1	4.25	5.29
ISSA-VMD	Tired	SWT-Soft	Tired	-1.37	47.57	<0.0001	1	4.81	5.86
ISSA-VMD	Tired	SWT-Hard	Easy	-0.80	59.44	<0.0001	1	6.15	7.19
ISSA-VMD	Tired	SWT-Hard	Transition	0.52	54.78	<0.0001	1	5.62	6.67
ISSA-VMD	Tired	SWT-Hard	Tired	-1.58	67.68	<0.0001	1	7.07	8.12
ISSA-VMD	Tired	ISSA-VMD	Easy	-2.82	29.73	<0.0001	1	2.81	3.86
ISSA-VMD	Tired	ISSA-VMD	Transition	-2.25	31.91	<0.0001	1	3.06	4.10
ISSA-VMD-SWT	Easy	SWT-Soft	Easy	-0.92	100.04	<0.0001	1	10.70	11.75
ISSA-VMD-SWT	Easy	SWT-Soft	Transition	-1.44	88.98	<0.0001	1	9.46	10.50

ISSA-VMD-SWT	Easy	SWT-Soft	Tired	2.67	94.02	<0.0001	1	10.03	11.07
ISSA-VMD-SWT	Easy	SWT-Hard	Easy	1.43	105.90	<0.0001	1	11.36	12.40
ISSA-VMD-SWT	Easy	SWT-Hard	Transition	2.00	101.24	<0.0001	1	10.84	11.88
ISSA-VMD-SWT	Easy	SWT-Hard	Tired	3.33	114.14	<0.0001	1	12.28	13.33
ISSA-VMD-SWT	Easy	ISSA-VMD	Easy	2.81	76.19	<0.0001	1	8.03	9.07
ISSA-VMD-SWT	Easy	ISSA-VMD	Transition	4.25	78.36	<0.0001	1	8.27	9.31
ISSA-VMD-SWT	Easy	ISSA-VMD	Tired	2.43	46.45	<0.0001	1	4.69	5.73
ISSA-VMD-SWT	Transition	SWT-Soft	Easy	1.19	118.79	<0.0001	1	12.81	13.85
ISSA-VMD-SWT	Transition	SWT-Soft	Transition	1.75	107.73	<0.0001	1	11.56	12.61
ISSA-VMD-SWT	Transition	SWT-Soft	Tired	3.08	112.77	<0.0001	1	12.13	13.17
ISSA-VMD-SWT	Transition	SWT-Hard	Easy	2.56	124.64	<0.0001	1	13.46	14.50
ISSA-VMD-SWT	Transition	SWT-Hard	Transition	4.01	119.99	<0.0001	1	12.94	13.98
ISSA-VMD-SWT	Transition	SWT-Hard	Tired	-0.24	132.89	<0.0001	1	14.39	15.43
ISSA-VMD-SWT	Transition	ISSA-VMD	Easy	6.01	94.93	<0.0001	1	10.13	11.17
ISSA-VMD-SWT	Transition	ISSA-VMD	Transition	4.77	97.11	<0.0001	1	10.37	11.42
ISSA-VMD-SWT	Transition	ISSA-VMD	Tired	5.34	65.20	<0.0001	1	6.79	7.84
ISSA-VMD-SWT	Transition	ISSA-VMD-SWT	Easy	6.67	18.74	<0.0001	1	1.58	2.62
ISSA-VMD-SWT	Tired	SWT-Soft	Easy	6.15	97.20	<0.0001	1	10.38	11.43
ISSA-VMD-SWT	Tired	SWT-Soft	Transition	7.59	86.14	<0.0001	1	9.14	10.19
ISSA-VMD-SWT	Tired	SWT-Soft	Tired	3.34	91.18	<0.0001	1	9.71	10.75
ISSA-VMD-SWT	Tired	SWT-Hard	Easy	3.58	103.05	<0.0001	1	11.04	12.08
ISSA-VMD-SWT	Tired	SWT-Hard	Transition	11.22	98.39	<0.0001	1	10.52	11.56
ISSA-VMD-SWT	Tired	SWT-Hard	Tired	9.98	111.29	<0.0001	1	11.96	13.01
ISSA-VMD-SWT	Tired	ISSA-VMD	Easy	10.55	73.34	<0.0001	1	7.71	8.75
ISSA-VMD-SWT	Tired	ISSA-VMD	Transition	11.88	75.52	<0.0001	1	7.95	8.99
ISSA-VMD-SWT	Tired	ISSA-VMD	Tired	11.36	43.60	<0.0001	1	4.37	5.41
ISSA-VMD-SWT	Tired	ISSA-VMD-SWT	Easy	12.81	2.85	0.68318	0	-0.84	0.20
ISSA-VMD-SWT	Tired	ISSA-VMD-SWT	Transition	8.55	21.59	<0.0001	1	-2.94	-1.90

*In the table, SEM value is 0.16 and Alpha is 0.05

Table S 6 Interactive grouping text table related Figure 1.

Denoising Method	State of fatigue	Mean value	Grouping								
ISSA-VMD-SWT	Transition	95.39	A								
ISSA-VMD-SWT	Easy	93.29		B							
ISSA-VMD-SWT	Tired	92.97		B							
ISSA-VMD	Tired	88.08			C						
ISSA-VMD	Easy	84.74				D					
ISSA-VMD	Transition	84.50				D					
SWT-Soft	Transition	83.31					E				
SWT-Soft	Tired	82.74						F			
SWT-Soft	Easy	82.07							G		
SWT-Hard	Transition	81.93							G		
SWT-Hard	Easy	81.41								H	
SWT-Hard	Tired	80.48									I

*The method of not sharing letters is significantly different.

Suppl. Figure

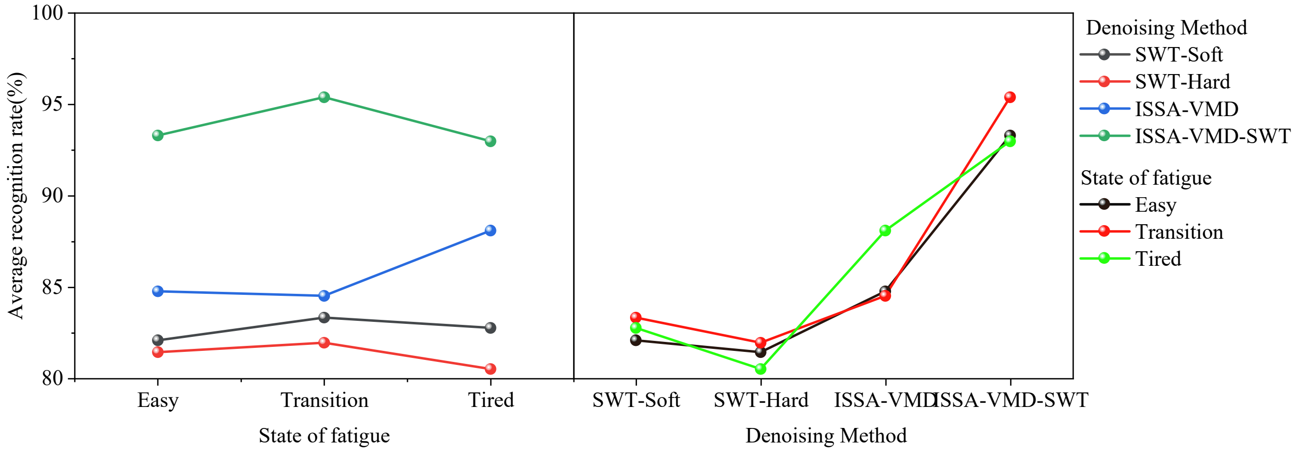

Fig.S 1 Interaction diagram of two-factor ANOVA, related Table 4.
